# Supplementary material for: In the Eye of the Storm: A Quantitative and Qualitative Account of the Impact of the COVID-19 Pandemic on Dutch Home Healthcare
Source: Int J Environ Res Public Health. 2022 Feb 16;19(4):2252. doi: 10.3390/ijerph19042252 (PMC8872342; doi:10.3390/ijerph19042252)
Supplement: Supplementary file 1 [file ijerph-19-02252-s001.zip › ijerph-1536898-supplementary.pdf]

SUPPLEMENTARY MATERIALS

- Analyses on trends in HHC use during the COVID-19 pandemic on Dutch HHC claims data
- Figure S1: Average HHC hours per client
- Figure S2: Changes in HHC intensity per client

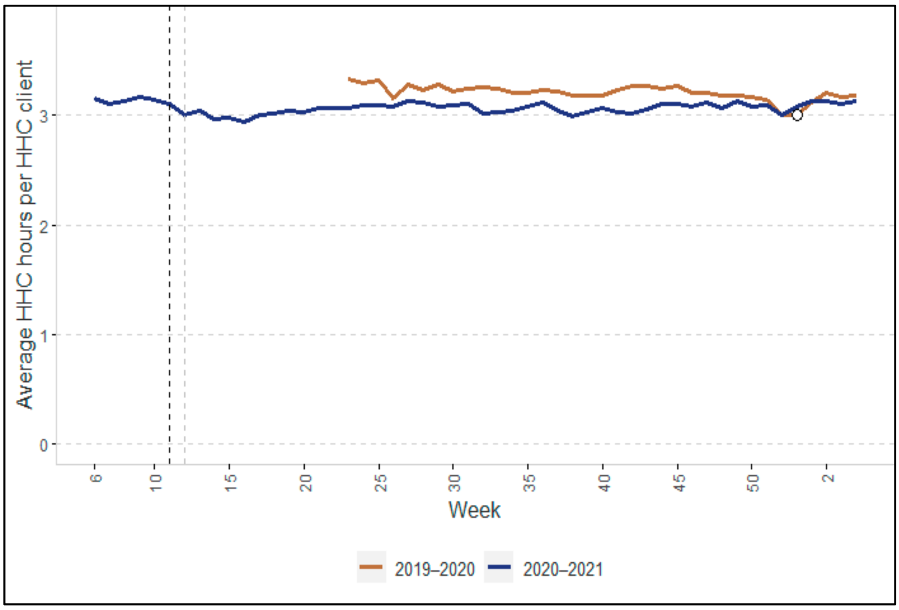

Figure S1. Average HHC hours per client.

Average HHC hours per client in 2020-2021, compared with the previous year (2019-2020).

Note: Because the year 2020 had 53 weeks rather than 52 weeks, we added a datapoint (the white dot) for week 53 for 2019 using the value of week 52.

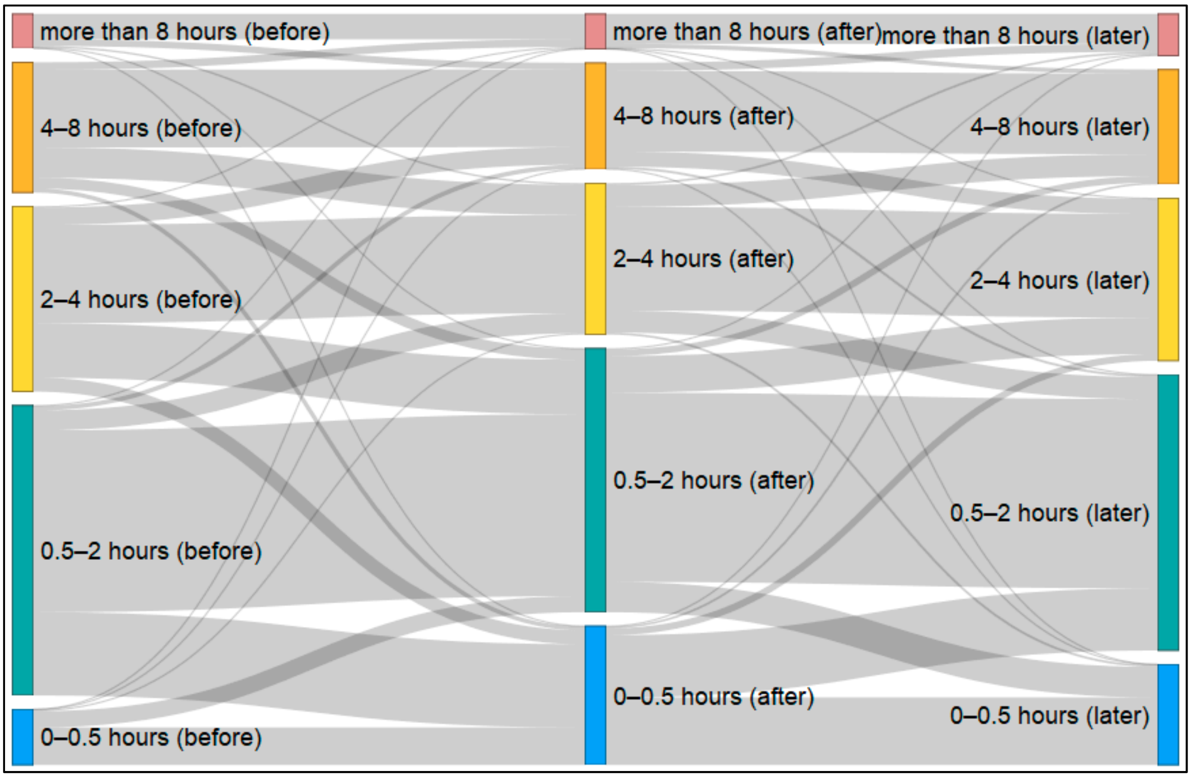

Figure S2. Changes in HHC intensity per client.

Average intensity of HHC in week 6-10 (left), week 13-17 (middle) and week 20-24 (right) in 2020, per client.

•

Analyses on trends in HHC use during the COVID-19 pandemic on Dutch HHC claims data

We used Dutch HHC claims data for the years 2019 and 2020 for this analysis. These data were not yet available during the study and therefore were not included in the study design and not taken into account in the focus group interviews. The claims dataset originates from the Dutch national information system of health insurers (Vektis) and includes all expenses that were claimed for the delivery of HHC in 2019 and 2020.

The dataset contains HHC use per month on the client level. For each client, gender and age are known. As we expect the summer and Christmas holidays to have an effect on HHC use, we created the dummies 'SummerHoliday' (for July and August) and 'ChristmasHoliday' (for December). To test the effect of COVID-19, we also included a dummy for April 2020 i.e. the month after the start of the COVID pandemic (variable 'MonthAfterCovid'). The table below provides a description of the data.

Table S1. Summary statistics.

Variable	*N*	*Mean*	*Std. Dev.*	*Min*	*Pctl. 25*	*Pctl. 75*	*Max*
Weighted HHC hours	6797395	913.75	1251.43	0	255	1156	83130
MonthAfter-Covid	6797395						
... 0	6529285	96%					
... 1	268110	4%					
SummerHoli-day	6797395						
... 0	5673083	83%					
... 1	1124312	17%					
ChristmasHoli-day	6797395						
... 0	6237529	92%					
... 1	559866	8%					
Age	6797395	78.02	14.37	0	73	87	110
Gender	6797395						
... 1	2513696	37%					
... 2	4283699	63%					
Year	6797395						
... 2019	3450422	51%					
... 2020	3346973	49%					

Visually, we see that throughout the year the total HHC hours provided fluctuates. In 2020, we observe bigger fluctuations than in 2019 and deviation from the 2019 trend, especially in April and May which are the months after the start of the COVID pandemic. This is in line with Figure 1 from the research article.

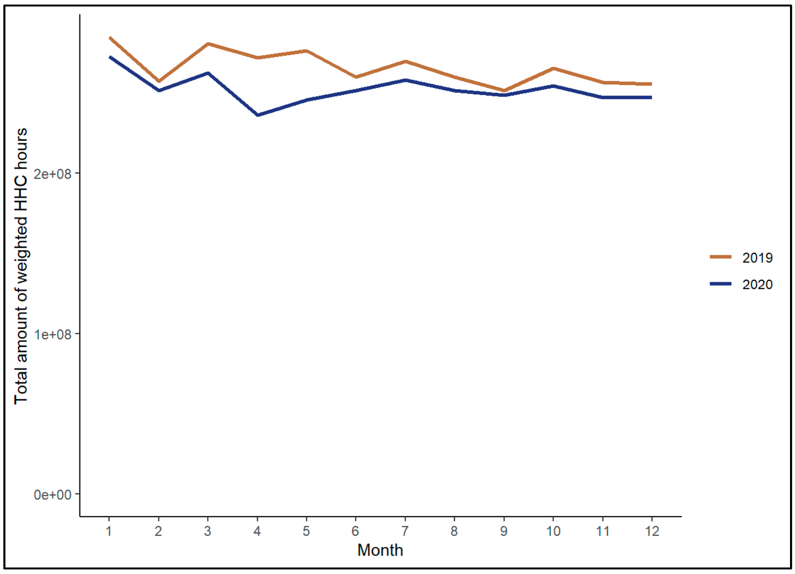

Figure S3. Weighted HHC hours per month.

We used negative binomial regression to test if the COVID-19 pandemic significantly impacted the HHC use in the Netherlands. Weighted HHC hours was our independent variable, and the other variables as provided in Table 1 were used as predictors. In the table below, the regression results are shown. We observe a significant negative effect of the COVID-19 pandemic (modelled as 'MonthAfterCovid') on HHC use.

Table S2. Regression results.

Term	*estimate*	*std.error*	*statistic*	*p.value*
(Intercept)	6.72077554	0.002260288	2973.415	0*
MonthAfterCovid1	-0.02891159	0.002066495	-13.991	1.78E-44*
SummerHoliday1	0.01007994	0.001068053	9.438	3.81E-21*
ChristmasHoliday1	-0.01742787	0.001435768	-12.138	6.61E-34*
Age	0.00081996	2.78E-05	29.512	1.99E-191*
Gender2	0.06688601	0.00082663	80.914	0*
Year2020	-0.01914054	0.000816942	-23.429	2.14E-121*

a. Note: * $p < 0.001$
